# The ASSIST Study - The BD Odon Device for assisted vaginal birth: a safety and feasibility study

**DOI:** 10.1186/s13063-019-3249-z

**Published:** 2019-03-05

**Authors:** Stephen O’Brien, Emily J. Hotton, Erik Lenguerrand, Julia Wade, Cathy Winter, Tim J. Draycott, Joanna F. Crofts, The ASSIST Study Group, Mary Alvarez, Sabaratnum Arulkumaran, Nichola Bale, Natalie Blencowe, Joanna F. Crofts, Timothy J. Draycott, Lily Exell, Anne Glover, Sally Hall, Emily J. Hotton, Erik Lenguerrand, Helen Lewis-White, Naomi Mallinson, Michelle Mayer, Sadie McKeown-Keegan, Glen Mola, Stephen O’Brien, Alison Pike, Iona Smith, Claire Rose, Sherrie Villis, Julia Wade, Paul White, Cathy Winter

**Affiliations:** 1 Translational Health Sciences, Bristol Medical School, University of Bristol, Bristol, UK; 2 Women & Children’s Directorate, North Bristol NHS Trust, Bristol, BS10 5NB UK; 3 Population Health Sciences, Bristol Medical School, University of Bristol, Bristol, UK

**Keywords:** BD Odon Device, Forceps, Ventouse, Assisted birth, Birth, Intrapartum research

## Abstract

**Background:**

Assisted vaginal birth is a vital health intervention that can result in better outcomes for mothers and their babies when complications arise in the second stage of labour. Unfortunately, instruments for assisted vaginal birth (forceps and ventouse) are often not utilised in settings where there is most clinical need, resulting in maternal and neonatal morbidity and mortality which could have been prevented. The BD Odon Device is a new device for assisted vaginal birth that may be able to address this unmet need. However, before dissemination, the device requires evaluation in robust clinical trials. A feasibility study to investigate the clinical impact, safety, and acceptability of the BD Odon Device for assisted vaginal birth is therefore planned. This will provide further information on acceptability, recruitment, and the outcome data required to design a future randomised controlled trial of the BD Odon Device versus Kiwi ventouse.

**Methods:**

Forty women who require an assisted vaginal birth for a recognised clinical indication will have the birth assisted with the BD Odon Device. The primary outcome is successful vaginal birth completed with the BD Odon Device. Secondary clinical outcomes include maternal and neonatal outcomes, and maternal and practitioner satisfaction. Safety data will be reviewed following every birth.

**Discussion:**

A future randomised controlled trial of the BD Odon Device versus the current standard instrument (the Kiwi ventouse) is planned. The findings of the ASSIST Study will inform the randomised controlled trial design.

**Trial registration:**

ISRCTN, ISRCTN10203171. Prospectively registered on 27 July 2018.

**Electronic supplementary material:**

The online version of this article (10.1186/s13063-019-3249-z) contains supplementary material, which is available to authorized users.

## Background

**Fig. 1:**
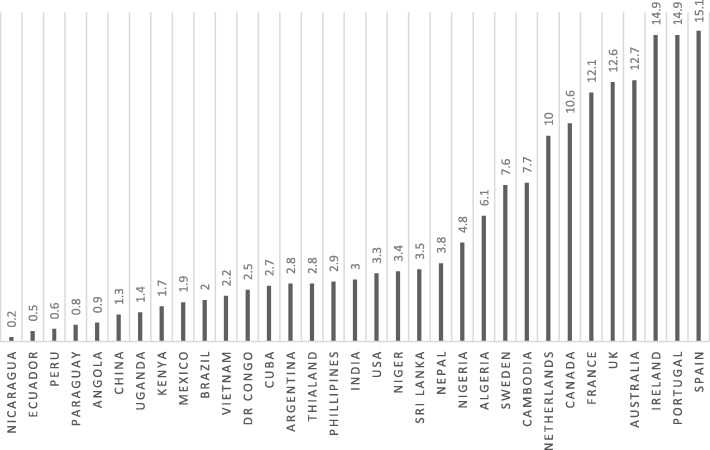
Percentage of births as AVBs in selected countries, 2008 to 2015. Data adapted from [18–21]

Complications of the second stage of labour (fetal compromise, obstructed labour, maternal exhaustion, or maternal medical conditions exacerbated by the act of pushing) remain a major cause of maternal and neonatal mortality and morbidity across the world. Such complications are responsible for 4% to 13% of maternal deaths in Africa, Asia, Latin America, and the Caribbean [1], and in 2013 obstructed labour alone accounted for four deaths per million women worldwide [2]. This burden of adverse outcomes may be reduced by an appropriately timed and safely performed assisted vaginal birth (AVB). An AVB is performed with either obstetric forceps or ventouse, and reduces adverse outcomes for women and their babies relative to caesarean section performed in the second stage of labour [3]. However, AVB rates are minimal in low- and middle-income countries (LMICs) where it is likely that there is the greatest need for AVB (Fig. 1).

In addition to widespread low levels of utilisation, earlier surveys report significant regions where AVB was not used at all—in 2006 this was the case in 74% of Latin American and Caribbean countries, as well as 30% of countries in sub-Saharan Africa and 40% of countries in Asia [4]. In addition to a lack of trained accouchers in LMICs [5–7], the maintenance and sterilisation requirements of both forceps and ventouse may limit their utilisation [4]. There is a significant unmet need for AVB in all maternity settings, but particularly in LMICs.

Forceps are less likely to fail in achieving an AVB when compared to a ventouse; however, they are associated with increased maternal perineal and vaginal trauma. The ventouse is less likely to achieve a vaginal birth and its use is associated with an increased risk of neonatal cephalohaematoma and retinal haemorrhage [8]. Both devices are efficient but do have caveats for their use. It is possible that a new device may be able to address some of the adverse events associated with the current devices used to assist birth.

**Fig. 2:**
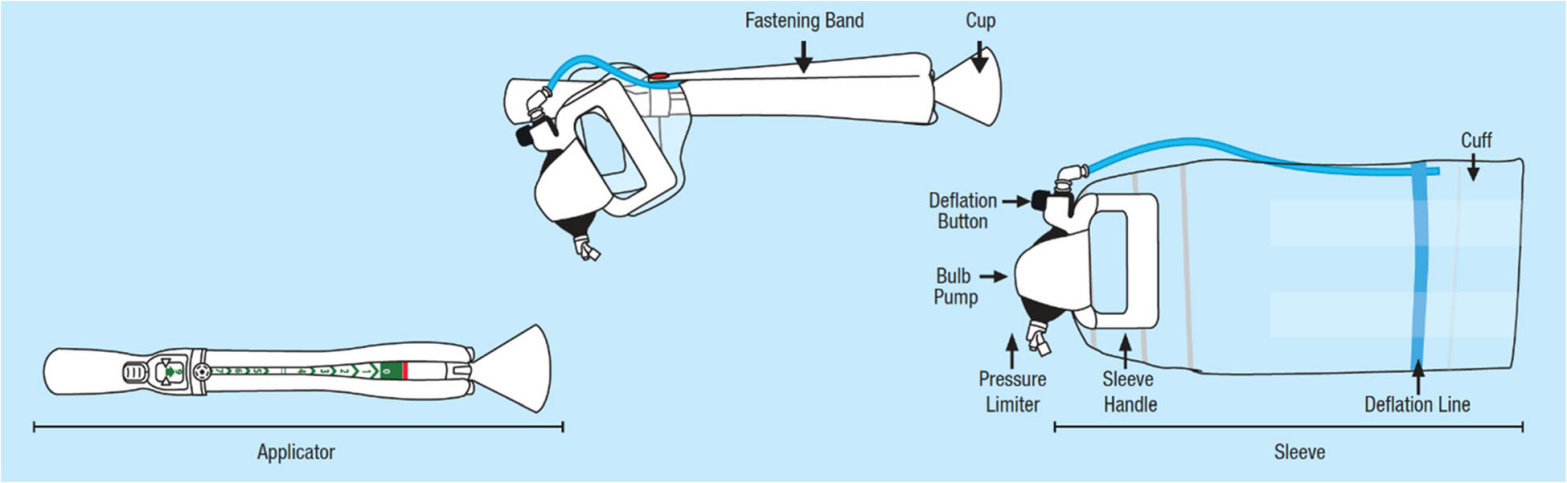
The BD Odon Device component parts

The BD Odon Device is a new device for AVB (Fig. 2). The use of an air chamber to act as the traction point on the fetal head (rather than the thin metal blades of the forceps) is hypothecated to reduce adverse events associated with the greater pressures applied to the fetal head during the use of forceps. The lack of negative pressure on the fetal head, the mechanism of action of the ventouse, obviates the risk of haematoma and haemorrhage associated with ventouse. Both of these contentions have been supported in pre-clinical simulation studies [9, 10]. A first-in-human pilot study of an earlier version of the device in healthy volunteers has been completed and demonstrated that assisting birth using the device is feasible [11]. Following the completion of extensive simulation studies which included human factor engineering validation testing, it has been deemed appropriate to evaluate the BD Odon Device in the intended target user population [12]. To date, the effectiveness and safety of the Odon device compared with other devices remains untested. It is now time to evaluate the clinical effectiveness and safety of the BD Odon Device in its intended clinical setting, using an appropriately powered and robust randomised controlled trial (RCT). To inform the design of this study we plan to conduct a safety and feasibility study exploring the clinical impact that the BD Odon device may have on current clinical practice, as well as the safety and acceptability of the device to women, midwives, obstetricians, and neonatologists.

## Methods/design

### Aim

This feasibility study will investigate the clinical impact, safety, and acceptability of the BD Odon Device and assess the feasibility of recruiting women and data collection. It will provide vital information on acceptability, recruitment, and the outcome data required to design a future RCT of the BD Odon Device versus the Kiwi ventouse.

### Study design

**Fig. 3:**
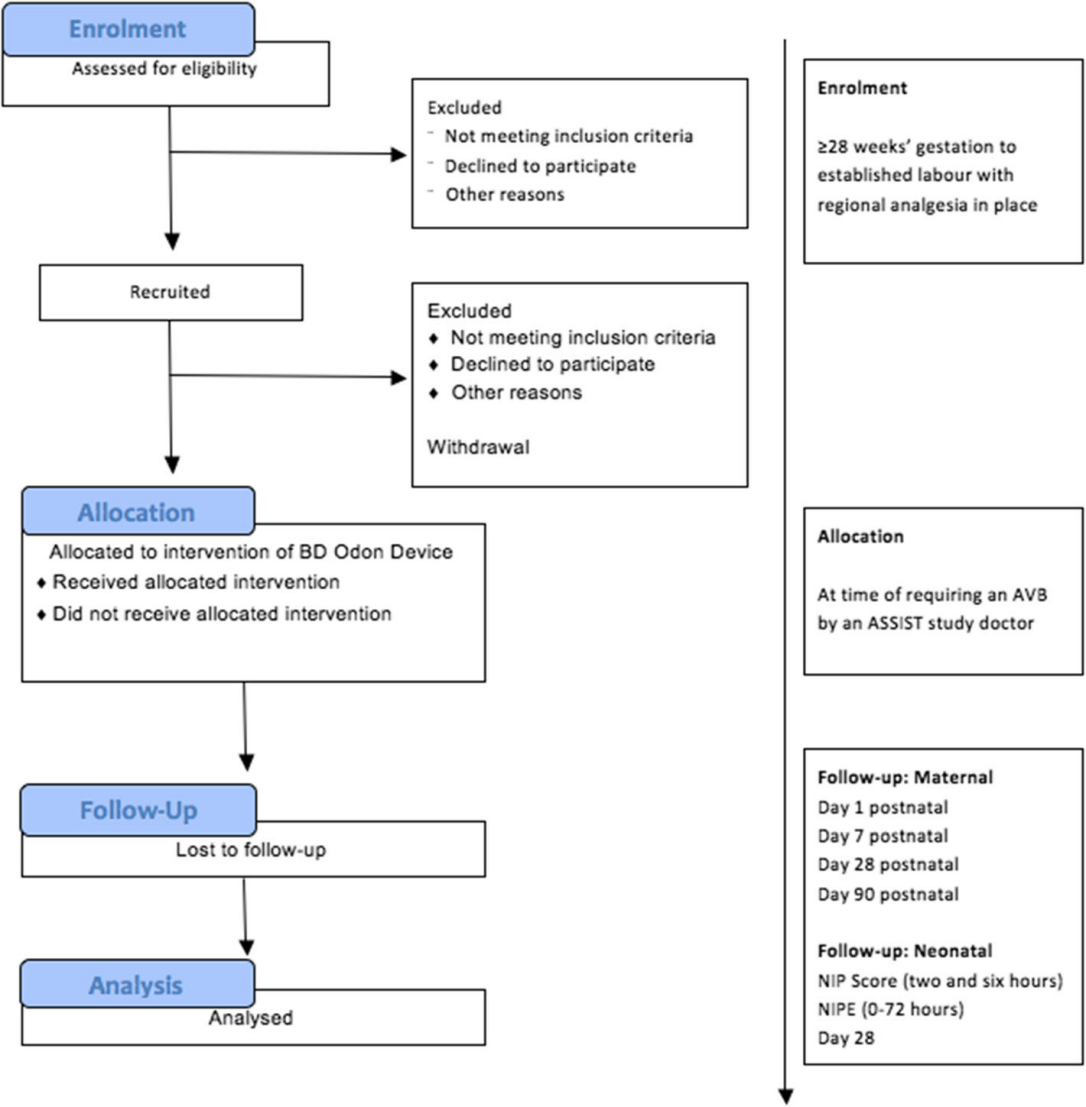
CONSORT diagram of the ASSIST feasibility study. AVB assisted vaginal birth, NIP Neonatal Infant Pain

The ASSIST Study (Assisted Vaginal Birth Study) is a non-randomised feasibility study of 40 women who require an assisted vaginal birth for a recognised clinical indication and who will all have their birth assisted with the BD Odon Device. A CONSORT diagram of the feasibility study is shown in Fig. 3.

The ASSIST feasibility study will utilise only one of the devices intended for use in the eventual randomised controlled study (BD Odon Device). This is to establish the safety, acceptability, and efficacy of the BD Odon Device prior to moving to a full RCT. The intended comparator (the Kiwi ventouse) will not be evaluated in this study since published evidence on its acceptability and success rate at the intended primary site already exists [13].

### Population/sample

Participants will be pregnant women aiming for a vaginal birth who plan to give birth at North Bristol NHS Trust (NBT), Bristol, UK. Recruitment is projected to continue for 8 months (due to the AVB rate within the department) after which time it is estimated that 40 sets of primary outcome data will have been recorded.

**Fig. 4:**
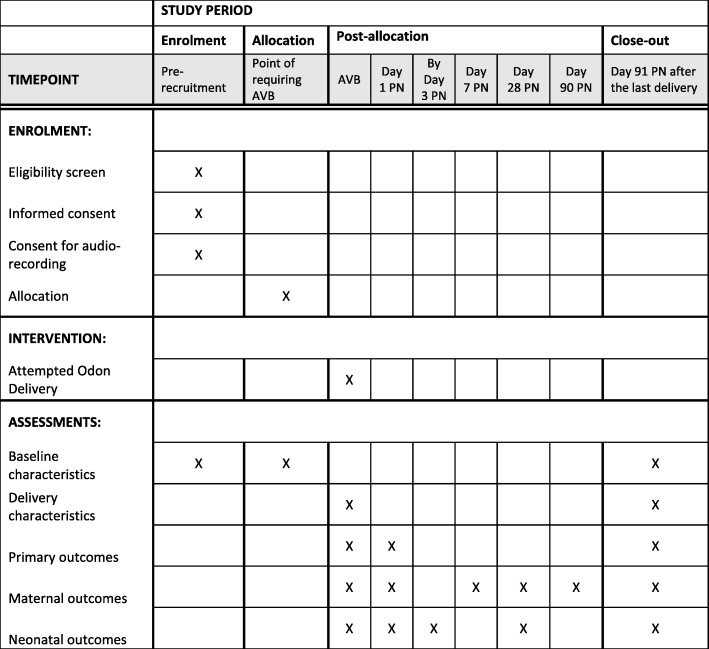
Schedule of enrolment, interventions, and assessments. AVB assisted vaginal birth, PN post-natal

Prospective participants will receive information on the study in early pregnancy (12 to 28 weeks) via the NBT Maternity ‘App’ (this ‘App’ is provided to all pregnant women at NBT and provides information on all aspects of their maternity care) and paper information leaflets given to women at any hospital admission. Members of the study team will then approach women after 28 completed weeks of pregnancy during antenatal appointments or antenatal admissions to discuss the study and offer women the opportunity to watch a video explaining the study. Women who are willing to take part (should they require an AVB) will then be invited to provide informed written consent. Figure 4 demonstrates the schedule of enrolment, interventions, and assessments.

When a woman who has previously consented to participate in the study arrives on the labour ward, her eligibility to participate in the study will be re-checked by a midwife and obstetrician that have been trained in Good Clinical Practice (GCP), and verbal re-confirmation of her consent to take part in the study will be sought by a GCP-trained midwife or obstetrician. Case report forms can be found in Additional file 1.

### Inclusion and exclusion criteria

Women will be able to participate in the ASSIST Study if all of the following apply at initial consent: the woman is ≥18 years of age; the woman has a singleton pregnancy of at least 36 weeks gestation; there is a negative antenatal screen for HIV and hepatitis B; the woman is in labour and requires an assisted vaginal birth for a clinical indication (as per the Royal College of Obstetrics and Gynaecology (RCOG) Greentop Guideline 26 [8]); the RCOG specific requirements for AVB are fulfilled; the woman has effective analgesia in place during the use of the instrument (i.e. epidural, spinal or pudendal block, or perineal infiltration with local anaesthetic); and there is no obstetric indication for an alternative method of AVB.

Women will not be able to take part in the ASSIST Study if: there is a diagnosis of a fetal skull abnormality precluding AVB (i.e. macrocephaly); there is a known osteogenesis imperfecta affected pregnancy; there is suspicion of a fetal bleeding disorder (von Willebrand’s disease, autoimmune thrombocytopenia (AITP), haemophilia); there is an intrauterine fetal death in the current pregnancy; the woman is sensitive to latex; the woman is currently serving a prison sentence; or the indication for AVB is a fetal bradycardia which is present, ongoing, and has not recovered.

### Intervention

If the obstetrician attending an eligible woman during the second stage of labour, and who has previously provided informed written consent to take part in the study, determines that an AVB is indicated, they will explain this to the woman as per standard practice. If the woman agrees to an AVB, an accoucheur who has had specific training in using the BD Odon Device (Additional file 2) will assist the birth with the BD Odon Device. Should the birth not be achieved with the BD Odon Device, the accoucheur will use their clinical judgement on an individual case basis to complete the birth using ventouse, forceps, or caesarean section as appropriate. Primary, secondary, safety, and qualitative research data will be gathered regarding the assisted birth and use of the device.

### Clinical, safety, and process outcomes

#### Primary outcome

The primary outcome will be the proportion of births successfully assisted with the BD Odon Device. A birth will be defined as ‘successful’ if all of the six criteria in Table 1 are met.

**Table 1:** Primary outcome

Criterion	Source	Collected by
The birth of the baby is expedited with the BD Odon Device	AVB pro-forma medical notes	Research team member including device operator
There are no serious maternal adverse events related to the use of the device during birth	AVB pro-forma medical notes	Research team member including device operator
There are no serious neonatal adverse events related to the use of the device during birth	AVB pro-forma medical notes	Research team member including device operator
There are no serious adverse device effects	AVB pro-forma medical notes	Research team member including device operator
The woman’s perception of her birth is rated above a score of 6	Case report form	Research team member
The practitioner reported outcome is above 12.	Case report form	Device operator

*AVB* assisted vaginal birth

#### Safety outcomes

The maternal safety outcomes are:

- Weighed/measured blood loss in the first 6 h following birth (post-partum haemorrhage ≥3000 ml);
- Third- or fourth-degree tear;
- Cervical tear requiring suturing;
- Requirement for general anaesthesia;
- Shoulder dystocia;
- Use of emergency caesarean section to achieve birth;
- Maternal death.

The neonatal safety outcomes are:

- Apgar score <7 at 5 min;
- Pressure necrosis of fat or skin;
- Neonatal soft tissue trauma (bruise/scalp/facial injury);
- Neonatal vascular injury (haemorrhage/cephalohaematoma/subaponeurotic haemorrhage);
- Neonatal skeletal injury (bone fracture);
- Neonatal intracranial injury (cerebral contusion);
- Neonatal neurological injury still present at 28 days after birth;
- Neonatal seizure;
- Phototherapy for jaundice contributed to by bruising;
- Death within 28 days after birth.

The device safety outcomes are:

- Failure of a component of the BD Odon Device;
- Number of applications of device;
- Number of pulls with the BD Odon Device.

#### Secondary outcomes

The clinical secondary outcomes are:

- Failure to achieve a vaginal birth with the assistance of the BD Odon Device and mode of birth thereafter;
- Method of infant feeding (day 1, 7, 28, and 90 post-natal);
- Time from ‘decision to perform assisted birth’ to ‘birth’ (minutes);
- Time from ‘device application’ to ‘birth’ (minutes);
- Time to achieve regular respirations (minutes);
- Episiotomy and perineal trauma;
- Umbilical arterial and venous pH and base excess;
- Other neonatal injury;
- Neonatal pain (Neonatal Infant Pain Score (NIPS) at 2 and 6 h after birth);
- Time spent in neonatal intensive care unit (hours);
- Anaemia requiring transfusion;
- Neonatal encephalopathy requiring therapeutic hypothermia within 28 days after birth;
- Organ failure within 28 days after birth;
- Failure to establish a normal feeding pattern, defined as ≤1 feed at 10 h of age.

The women-reported secondary outcomes are:

- Maternal health-related quality of life data (by EQ-5D-5 L antenatally at the time of consent, at day 1, and day 28 post-natal);
- Maternal satisfaction with birth experiences (Patient Perception Score on day 1 post-natal);
- Maternal perception of pain (day 1, 7, and 28 post-natal);
- Health service utilisation will be collected (day 28 post-natal);
- Maternal continence at 90 days.

The practitioner-reported secondary outcomes are:

- Willingness to use the BD Odon Device;
- Perceived overall ease of use of device;
- Ease of device set-up;
- Ease of device application to the baby’s head;
- Ease of withdrawal of the applicator after application;
- Comfort with the level of force required to assist the birth of the baby;
- Ease of deflation of the air chamber prior to crowning.

### Safety of the intervention

A comprehensive assessment of the safety of the BD Odon Device will be undertaken following every attempted birth within the ASSIST Study. Outcome measures and data collected will ensure capture of any potential adverse events associated with the BD Odon Device at the time of birth by the operator and/or a member of the research team (see Additional file 1 for details of case report forms). Follow-up will be performed by the research team. In the immediate post-partum period, a member of the research team will follow-up the participant on a daily basis until discharge, collecting the day 1 data. To ensure that any serious adverse events that occur in the post-natal period are captured, an Adverse Event Reporting System will be initiated on the post-natal wards for ward staff to highlight any serious adverse events that occur after discharge from the labour ward. Post-natal staff will be asked to notify a member of the research team if any such events occur. In addition, device failure (or failure of any component) will be reported as an individual outcome measure. Follow-ups at day 7, 28, and 90 will be conducted by a member of the research team by telephoning the woman using their contact details provided to the study team. The Trial Management Group (TMG) and Sponsor will regularly review data from all births according to the schedule in Additional file 3 to ensure early identification of any trends of adverse events. All adverse events will be classified and reported according to the schedule of the Medicines and Healthcare Regulation Agency (MHRA) and the Research Ethics Committee.

#### Recruitment and process outcomes

To facilitate the development of the main trial, information will be collected on the number of women screened, those identified as eligible and approached, and those who consented to participate both in advance of labour and again during labour where applicable. Information on data completion, i.e. questionnaire completion, completion of main outcomes, and missing data, will also be reported. Reasons for ineligibility, participation refusal, loss to follow-up, or missing data will also be explored.

### Patient and public involvement

Women and their partners have been involved throughout the development of the ASSIST Study. Formal patient and public involvement (PPI) panels have reviewed the proposed study design, patient-facing documentation (leaflets, videos, and consent forms), and have supported both the general and specific aims of the study. The Trial Steering Committee (TSC) of the ASSIST Study includes a lay patient representative.

### Sample size and analysis

A complete sample size of 40 women will enable the estimation of the rate of successful assisted vaginal birth of 80% to within a 95% confidence interval of ±12%. The sample size will also demonstrate AVB requiring use of a secondary instrument of 50% to within a 95% confidence interval of ±15%.

### Quantitative description

The primary, secondary, and safety data will be reported as the frequency and proportion, mean (and standard deviation), or median (and interquartile range) depending on their nature and distribution. The overall rate of successful AVB birth will be reported and the frequency of unsuccessful births will be reported by the six criteria defined for success. Appropriate outcomes will be presented and broken down into subgroups by operator experience (>10 years or <10 years), indication for assisted vaginal birth (fetal compromise or other indication of AVB), participant analgesia (regional anaesthetic or other analgesia), fetal station (0 and +1 cm to spines or +2 and +3 cm to spines), and fetal position (OA or OT or OP). The overall number of safety events will be reported, as well as the number of events by the main reason for adverse events. Events related to device failure and/or mis-use of the device will also be described.

The recruitment and process outcomes will be reported as frequencies and percentages. Completion rates of the clinical outcomes will also be reported. Reasons for ineligibility, refusal, loss to follow-up, or missing data will be categorised and described as frequencies.

Data on numbers screened, those identified as eligible and approached, those consenting to participate both in advance of labour and again during labour where applicable, those included in follow-up and those providing questionnaire and outcome data and successful birth rates will all inform the sample size calculation of the main trial. During the trial, there will be a continuous review of mother and baby safety from the Sponsor and TMG, and a decision will be made as to whether to continue, revise, or stop the trial.

Women and their babies will be followed up at 1 day, 7 days, 28 days, and 90 days following the birth. A woman and her baby will be deemed to complete their participation in the study at 90 days after the birth.

### Integrated qualitative research

Alongside the primary clinical study, an integrated qualitative study (IQS) within the feasibility study will be undertaken. This will investigate: the practitioners’ use of the device to ensure that an appropriate training package is developed for the trial; enable the intervention to be described and refined to optimise its use; and to investigate the woman’s, obstetrician’s, midwife’s, and neonatologist’s perspective of the birth and what they consider to be characteristics of a ‘good birth’. This will enable the research team to incorporate these findings into any subsequent RCT, as well as iteratively altering the ASSIST Study plan if required.

The qualitative study will combine observational and interview data collection and analysis. AVBs involving successful or attempted use of the BD Odon device will be observed wherever possible. Successful or attempted births will be followed up with interviews with obstetricians, midwives, and women to triangulate their experiences and views with what has been observed.

### Trial oversight

A TMG consisting of all investigators and co-investigators will be responsible for the day-to-day running of the study.

The study will be overseen by two committees, the Independent Data Monitoring Committee (IDMC) and the Trial Steering Committee (TSC). The IDMC will sit after 20 and 40 births have been completed and will have no direct involvement in the running of the trial. Following both reviews, the IDMC will generate a report on the performance of the device and the safety of participants. These reports will be reviewed by the TSC and the Sponsor. The TSC will consist of an independent clinical expert, statistician, and a lay representative, together with the investigators and representative. The TSC will review all reports produced by the IDMC and make a recommendation to the Sponsor following every review to continue, modify, or halt the study. The TSC will provide oversight of the progress of the study and ensure the study is conducted according to the principles of GCP. Auditing will take place when requested by the Sponsor.

### Dissemination

Study results will be published within 1 year of completion of data collection in an appropriate peer-reviewed, open-access journal. The results will be presented at local, national, and international meetings. Summaries will also be distributed using existing parent networks. A summary of results will also be sent to all women who participated in the study, unless they express their wish not to receive such information. Results will be communicated to a lay audience by social media activities of North Bristol NHS Trust, the University of Bristol, and the research team.

## Discussion

An appropriately conducted AVB, performed when clinically indicated, is associated with improved maternal and neonatal outcomes when compared with caesarean section in the second stage of labour or compared with no action [3]. AVB is currently not performed in many low- and middle-income settings where it may be of significant benefit to individual woman and their babies in reducing birth-related morbidity and mortality [14]. Current efforts to promote the use of AVB in these settings have been insufficiently effective which may be due to the inherent limitations of the existing instruments. Therefore, the development of a new device for AVB is both justified and required [15].

The BD Odon Device is the first new device for AVB since the introduction of ventouse into clinical practice in the 1950s [16]. Extensive pre-clinical simulation testing has suggested that it is not likely to generate additional pressure over the fetal head compared with current instruments [9, 17], is not likely to generate clinically significant levels of neonatal hypoxia if misplaced over the fetal carotid arteries [10], and is not likely to be associated with unsafe patterns of use by the target user population [12]. We believe that it is therefore reasonable to proceed to a clinical feasibility study of the device, and, if positive, a randomised controlled trial.

If found safe by the TSC, IDMC, and Sponsor, findings from this feasibility study will inform the design of a randomised controlled trial that may produce evidence that supports the introduction into clinical practice of a new device for AVB. If this were able to address the unmet need for AVB around the world it would have a major impact on the management of complications in the second stage of labour and maternal and neonatal outcomes worldwide.

## Trial status

The ASSIST Study is scheduled to commence on 8 October 2018, using version 16.10. The study will cease after 40 complete sets of primary outcome data are received. This is projected to be in May 2019.

## Additional files

Additional file 1: Case report forms. Copies of the case report forms for the ASSIST Study. (ZIP 830 kb)

Additional file 2: Operator training for the ASSIST Study. Details of the training programme operators received prior to study start. (DOCX 73 kb)

Additional file 3: Schedule of review of data collected in the ASSIST Study. Details of the Sponsor/TSC/IDMC reviews. (DOCX 96 kb)

Additional file 4: Grant agreement. Summary agreement and signature page from funder. (PDF 147 kb)

Additional file 5: SPIRIT 2013 checklist: recommended items to address in a clinical trial protocol and related documents. (DOC 120 kb)

Additional file 6: Ethics Committee Approval for the ASSIST Study format. Letter from the South Central Berkshire Ethics Committee. (PDF 372 kb)

## Abbreviations

AVB: Assisted vaginal birth
BD: Becton Dickinson
GCP: Good Clinical Practice
IDMC: Independent Data Monitoring Committee
IQS: Integrated qualitative study
LMIC: Low- and middle-income country
MHRA: Medicines and Healthcare Regulatory Agency
NBT: North Bristol NHS Trust
NIPS: Neonatal Infant Pain Score
PPI: Patient and public involvement
RCOG: Royal College of Obstetrics and Gynaecology
RCT: Randomised controlled trial
TMG: Trial Management Group
TSC: Trial Steering Committee
UoB: University of Bristol
