# Supplementary material for: The ASSIST Study - The BD Odon Device for assisted vaginal birth: a safety and feasibility study
Source: Trials. 2019 Mar 5;20:159. doi: 10.1186/s13063-019-3249-z (PMC6402154; doi:10.1186/s13063-019-3249-z)
Supplement: Supplementary file 1 — Case report forms. Copies of the case report forms for the ASSIST Study. (ZIP 830 kb) [file 13063_2019_3249_MOESM1_ESM.zip › PublicationFiles-supplementary_file_a1_R0.pdf]

NHS North Bristol NHS Trust

Please stick hospital identification label here

ASSIST ASSISTED VAGINAL BIRTH STUDY

Study number: __ __ __ __

MATERNAL CASE REPORT FORM A

PART 1: Clinical staff to complete on or prior to admission to labour ward

**Re-confirmation of consent (please tick)**<br>**Note: women are only eligible for inclusion if all answers fall within unshaded boxes**		
	**YES**	**NO**
Has consent been taken and documented?		(shaded)
Date consent received?	_ _/_ _/_ _	
Have you verbally re-confirmed consent and documented this in the partogram?		(shaded)
**Re-confirmation of Eligibility criteria (please tick)**<br>**Note: women are only eligible for inclusion if all answers fall within unshaded boxes**		
	**YES**	**NO**
At least 18 years of age?		(shaded)
Singleton pregnancy?		(shaded)
Gestation ≥ 36+0 weeks?		(shaded)
The woman may require an AVB?		(shaded)
Negative antenatal screen for HIV and Hepatitis B?		(shaded)
Is there is a confirmed diagnosis of a fetal skull abnormality precluding AVB (i.e. macrocephaly)?	(shaded)	
Is this a known osteogenesis imperfecta affected pregnancy?	(shaded)	
There is suspicion of a fetal bleeding disorder (von Willebrand's disease, ITP, haemophilia etc.)?	(shaded)	
Intrauterine fetal death in this pregnancy?	(shaded)	
Is the woman currently serving a prison sentence?	(shaded)	
Is the woman sensitive to latex?	(shaded)	
Does the woman have a lack of ability to read or understand English?<br>(As this would preclude successful completion of questionnaires)	(shaded)	

**Midwife sign when eligibility and consent has been re-confirmed**<br>**Name (PRINT):**<br>**Signature:**<br>**Date:**	**GCP trained doctor sign eligibility and consent has been re-confirmed**<br>**Name (PRINT):**<br>**Signature:**<br>**Date:**

Decision for AVB with Odon Device

Persistent fetal bradycardia is a contraindication to the use of the BD Odon Device in this study. Delivery should be expedited as soon as possible in line with routine clinical practice

GCP trained, approved ASSIST study doctor to sign and confirm consent, eligibility and decision

Name (PRINT): **Signature:**

Date: **Time of decision:**

**Birth Summary (please tick or circle)**				
**Primary indication for assisted birth (circle one)**	Presumed fetal compromise	Delay in 2nd stage	Maternal exhaustion	Maternal medical indication

**Did this woman have an attempted assisted vaginal birth with the BD Odon Device?**	
**Yes** ☐	**No** ☐
	**If NO, please note all that apply and number in order of sequence please:**
	Spontaneous vaginal delivery ☐
	Ventouse ☐
	Forceps ☐
	Emergency Caesarean section ☐

Please affix BD Odon Device ID number label
Ensure additional device ID number label is affixed to the birth notes and neonatal pro forma

PART 2: Clinical variable characteristics<br>Obstetrician to complete immediately post birth						
**Maternal age**		**BMI at booking**		**BMI at 36/40**		
**Maternal ethnicity (circle)**	White British	Black African	Bangladeshi	Chinese		
	White Irish	Black Caribbean	Indian	Any other Asian background		
	Any other white background	Any other black background	Pakistani	Variety of mixed backgrounds		
**EDD**						
**Gravidity & Parity**	G:	P:	# prev C-sections:			
**Onset of labour**	Spontaneous	Induced				
**Length of labour (hours & minutes)**	First stage<br>__ __:__ __	Second stage<br>__ __:__ __				
**Gestation at birth**	Weeks:	Days:				
**Date of baby's birth**	__ __/ __ __/ __ __					
**Birth weight (grams)**	__ __ __ __ g					
**Baby's head circumference (cm)**	__ __ cm					
**Position of fetal head (circle)**	ROA	OA	LOA			
	ROT		LOT			
	ROP	OP	LOP			
**Head Palpable per abdomen**	0/5	1/5				
**Station**	At spines	+1	+2	+3		
**USS used to determine station?**	Yes	No				
**Caput**	None	1cm	2cm	3cm		
**Moulding**	None	+	++	+++		
**Analgesia**	Epidural	Spinal	Pudendal	Perineal infiltration	GA	None
**Grade of operator**	ST 3 - 5	ST 6 - 7	Cons			

<table>
<tr><th colspan="7">PART 3: Maternal short-term outcomes
Obstetrician to complete prior to discharge from CDS</th></tr>
<tr><td>Time of events</td><td colspan="2">Time of decision:
__ __:__ __</td><td colspan="2">Time of first device application:
__ __:__ __</td><td colspan="2">Time of birth:
__ __:__ __</td></tr>
<tr><td>Location of birth</td><td colspan="3">Delivery Suite Room</td><td colspan="3">Operating Theatre</td></tr>
<tr><td>Number of pulls with Odon Device?</td><td colspan="2"></td><td colspan="2">Number of applications the Odon Device?</td><td colspan="2"></td></tr>
<tr><td>Episiotomy performed?</td><td colspan="3">Yes</td><td colspan="3">No</td></tr>
<tr><td>Success with Odon Device?</td><td colspan="3">Yes</td><td colspan="3">No</td></tr>
<tr><td rowspan="4">If primary instrument FAILURE, please state your perceived reason(s)

(circle all appropriate and provide details)</td><td>Maternal</td><td colspan="5">e.g. lack of cooperation, exhaustion, poor analgesia</td></tr>
<tr><td>Device</td><td colspan="5">e.g. device faulty, unable to use safely, slippage of device on fetal head</td></tr>
<tr><td>Clinical/ Situational</td><td colspan="5">e.g. inappropriate equipment, lack of theatre staff</td></tr>
<tr><td>Other</td><td colspan="5"></td></tr>
<tr><td>Second instrument used at AVB?</td><td colspan="2">Yes</td><td colspan="4">No</td></tr>
<tr><td>If yes, which instrument</td><td>Kiwi ventouse</td><td>Silastic ventouse</td><td>Forceps</td><td colspan="3">Other State:_______________</td></tr>
<tr><td>Caesarean section performed?</td><td colspan="2">Yes</td><td colspan="4">No</td></tr>
<tr><td>Cervical tear present?</td><td>Yes</td><td>No</td><td colspan="2">Does the tear require suturing?</td><td>Yes</td><td>No</td></tr>
<tr><td>Ischio-rectal fossa defect present?</td><td colspan="3">Yes</td><td colspan="3">No</td></tr>
<tr><td>Is there a labial tear requiring suturing</td><td colspan="3">Yes</td><td colspan="3">No</td></tr>
<tr><td>Degree of perineal trauma</td><td>None</td><td>1st degree</td><td>Episiotomy</td><td>2nd degree</td><td>3rd degree:
A B C</td><td>4th degree</td></tr>
<tr><td>Weighed EBL (ml)</td><td colspan="6">__ __ __ __ ml</td></tr>
</table>

**PART 4: Practitioner reported outcomes – Obstetrician to complete prior to discharge from CDS**					
**Perceived overall ease of use of device**	Very difficult<br>1	2	3	4	Very easy<br>5
**Ease of instrument set-up**	Very difficult<br>1	2	3	4	Very easy<br>5
**Ease of instrument application to the baby's head**	Very difficult<br>1	2	3	4	Very easy<br>5
**Ease of withdrawal of applicator after application**	Very difficult<br>1	2	3	4	Very easy<br>5
**Comfort with the level of force required to deliver the baby**	Very difficult<br>1	2	3	4	Very easy<br>5
**Ease of deflation of the air chamber prior to crowning**	Very difficult<br>1	2	3	4	Very Easy<br>5
**Any other comments or impressions regarding the birth with the Odon Device?**	*Please insert free comments below:*				

**Initial Adverse Event Reporting Tool *see appendix X**<br>**Member of research team to complete (photocopy p.4&5 to sponsor)**	
**Event Description**	TICK HERE **IF THIS IS A RED EVENT**: ☐
**Situation**	
**Background**	
**Action**	
**Assessment undertaken by:**	**Name:**<br>**Signature:**<br>**Date:**

PART 5: Maternal medium-term outcomes – Research staff to complete on day 1		
**Date collected:**		
**Has the women required any analgesia in the last 6 hours?**	Yes	No
**If yes the please tick all analgesia required in the first 24 hours following birth (not including analgesia for birth, if applicable) and total dose:**	☐ Paracetamol<br>☐ Ibuprofen<br>☐ Dihydrocodeine<br>☐ Codeine<br>☐ Diclofenac<br>☐ Oramorph<br>☐ Other……………………….<br>…………………………………….<br>………………………………………	………..…………………………g<br>…………………………………mg<br>…………………..……………mg<br>……………………..………..mg<br>…………………….………..mg<br>…………………….………..mg<br>…………………….……………….<br>……………………………………..<br>……………………………………..

**Women's perception of birth**						
**Question**	**Strongly Disagree**	**Disagree**	**Neutral**	**Agree**	**Strongly Agree**	**Score**
**I felt well informed due to good communication**	**1**	**2**	**3**	**4**	**5**	
**I felt I was treated with respect at all times**	**1**	**2**	**3**	**4**	**5**	
**I felt safe at all times**	**1**	**2**	**3**	**4**	**5**	
					**Total**	

**Women's perception of pain**<br>**Please circle number indicating current level of pain**										
**No pain at all**										**Extremely severe pain**
**1**	**2**	**3**	**4**	**5**	**6**	**7**	**8**	**9**	**10**	**11**

**Did the baby have at least one good feed at or before 10 hours old?**	**Yes**	**No**

**How are you currently feeding your baby?**		
**Breast**	**Mixed**	**Bottle**

Study number: ___ ___ ___ ___

The best health you can imagine

Under each heading, please tick the ONE box that best describes your health TODAY	
**MOBILITY**	
I have no problems in walking about	❑
I have slight problems in walking about	❑
I have moderate problems in walking about	❑
I have severe problems in walking about	❑
I am unable to walk about	❑
**SELF-CARE**	
I have no problems washing or dressing myself	❑
I have slight problems washing or dressing myself	❑
I have moderate problems washing or dressing myself	❑
I have severe problems washing or dressing myself	❑
I am unable to wash or dress myself	❑
**USUAL ACTIVITIES** *(e.g. work, study, housework, family or leisure)*	
I have no problems doing my usual activities	❑
I have slight problems doing my usual activities	❑
I have moderate problems doing my usual activities	❑
I have severe problems doing my usual activities	❑
I am unable to do my usual activities	❑
**PAIN / DISCOMFORT**	
I have no pain or discomfort	❑
I have slight pain or discomfort	❑
I have moderate pain or discomfort	❑
I have severe pain or discomfort	❑
I have extreme pain or discomfort	❑
**ANXIETY / DEPRESSION**	
I am not anxious or depressed	❑
I am slightly anxious or depressed	❑
I am moderately anxious or depressed	❑
I am severely anxious or depressed	❑
I am extremely anxious or depressed	❑

Study number: ___ ___ ___ ___

We would like to know how good or bad your health is TODAY.

This scale is numbered from 0 to 100.

100 means the best health you can imagine.
0 means the worst health you can imagine.

Mark an X on the scale to indicate how your health is TODAY.

Now, please write the number you marked on the scale in the box below.

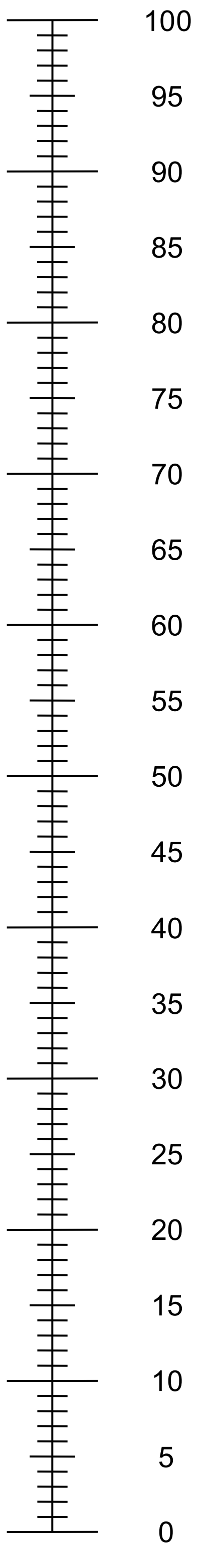

YOUR HEALTH TODAY = [ ]

The worst health
you can imagine
