# Supplementary material for: The ASSIST Study - The BD Odon Device for assisted vaginal birth: a safety and feasibility study
Source: Trials. 2019 Mar 5;20:159. doi: 10.1186/s13063-019-3249-z (PMC6402154; doi:10.1186/s13063-019-3249-z)
Supplement: Supplementary file 1 — Case report forms. Copies of the case report forms for the ASSIST Study. (ZIP 830 kb) [file 13063_2019_3249_MOESM1_ESM.zip › PublicationFiles-supplementary_file_a2R0.pdf]

North Bristol NHS Trust

Participant ID: ___ ___ ___ ___

ASSIST
ASSISTED VAGINAL BIRTH STUDY

Please attach hospital identification label

MATERNAL CASE REPORT FORM B

PART 1: Maternal long-term outcomes – Research staff to complete on day 7		
**Date collected:**		
**Has the women required any analgesia in the last 24 hours?**	Yes	No
**If so, what? (record all drugs and doses)**	☐ Paracetamol<br>☐ Ibuprofen<br>☐ Dihydrocodeine<br>☐ Codeine<br>☐ Diclofenac<br>☐ Oramorph<br>☐ Other……………………………<br>……………………………………<br>……………………………………<br>……………………………………	………………………………………g<br>………………………………………mg<br>………………………………………mg<br>………………………………………mg<br>………………………………………mg<br>………………………………………mg<br>……………………………………<br>……………………………………<br>……………………………………<br>……………………………………

**Women's perception of pain**<br>**Please circle number indicating current level of pain**										
**No pain at all**										**Extremely severe pain**
**1**	**2**	**3**	**4**	**5**	**6**	**7**	**8**	**9**	**10**	**11**

**How are you currently feeding your baby?**		
**Breast**	**Mixed**	**Bottle**

Participant ID: ___ ___ ___ ___

PART 2: Maternal long-term outcomes – Research staff to complete on day 28		
**Date collected:**		
**Has the women required any analgesia in the last 24 hours?**	Yes	No
**If so, what? (record all drugs and doses)**	☐ Paracetamol<br>☐ Ibuprofen<br>☐ Dihydrocodeine<br>☐ Codeine<br>☐ Diclofenac<br>☐ Oramorph<br>☐ Other………………………….<br>…………………………………………<br>…………………………………………<br>…………………………………………	……………………………………g<br>……………………………………mg<br>……………………………………mg<br>……………………………………mg<br>……………………………………mg<br>……………………………………mg<br>…………………………………………<br>…………………………………………<br>…………………………………………<br>…………………………………………

**Women's perception of pain**<br>**Please circle number indicating current level of pain**										
**No pain at all**										**Extremely severe pain**
**1**	**2**	**3**	**4**	**5**	**6**	**7**	**8**	**9**	**10**	**11**

**How are you currently feeding your baby?**		
**Breast**	**Mixed**	**Bottle**

Please remember to perform EQ-5D-5L overleaf

Participant ID: ___ ___ ___ ___

Under each heading, please tick the ONE box that best describes your health TODAY	
**MOBILITY**	
I have no problems in walking about	☐
I have slight problems in walking about	☐
I have moderate problems in walking about	☐
I have severe problems in walking about	☐
I am unable to walk about	☐
**SELF-CARE**	
I have no problems washing or dressing myself	☐
I have slight problems washing or dressing myself	☐
I have moderate problems washing or dressing myself	☐
I have severe problems washing or dressing myself	☐
I am unable to wash or dress myself	☐
**USUAL ACTIVITIES** *(e.g. work, study, housework, family or leisure)*	
I have no problems doing my usual activities	☐
I have slight problems doing my usual activities	☐
I have moderate problems doing my usual activities	☐
I have severe problems doing my usual activities	☐
I am unable to do my usual activities	☐
**PAIN / DISCOMFORT**	
I have no pain or discomfort	☐
I have slight pain or discomfort	☐
I have moderate pain or discomfort	☐
I have severe pain or discomfort	☐
I have extreme pain or discomfort	☐
**ANXIETY / DEPRESSION**	
I am not anxious or depressed	☐
I am slightly anxious or depressed	☐
I am moderately anxious or depressed	☐
I am severely anxious or depressed	☐
I am extremely anxious or depressed	☐

The best health you can imagine

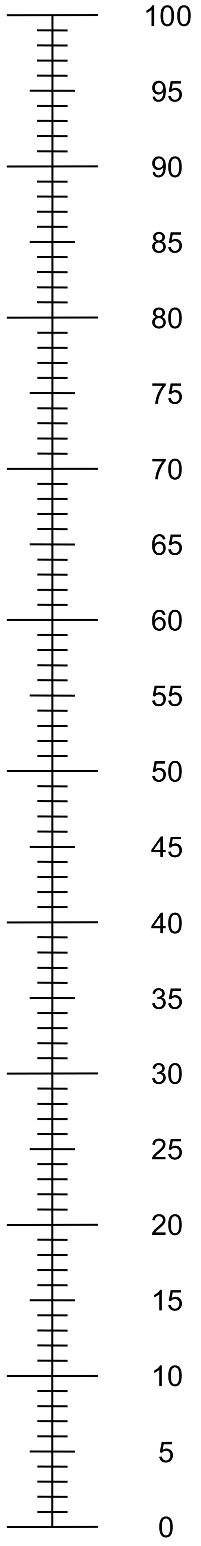

The worst health you can imagine

We would like to know how good or bad your health is TODAY.

This scale is numbered from 0 to 100.

100 means the best health you can imagine.

0 means the worst health you can imagine.

Mark an X on the scale to indicate how your health is TODAY.

Now, please write the number you marked on the scale in the box below.

YOUR HEALTH TODAY = [ ]

PART 3: Maternal long-term outcomes – Research staff to complete on day 90						
**Date collected:**						
**At present, do you ever lose any urine when you don't mean to?**						
Yes			No			
**In the past month, how often has this happened on average?**						
less than twice a month	twice a month	once a week	a few times a week	once a day	twice a day	three or more times a day
**Do you ever lose control of wind or bowel motions from your back passage between visits to the toilet?**						
no	rarely	sometimes	often	always		
**How are you feeling generally?**						
very well	well	not very well	not at all well			
**Do you lose urine when you: (yes/no)**						
Stress: cough, laugh, sneeze, run, jump, or play sport (if not also urge or mixed) **YES / NO**	Urge: feel an urgent desire to pass water and are unable to reach the toilet in time (if not also stress or mixed) **YES / NO**	Mixed: At some other time (including women who ticked both stress and urge) **YES / NO**				
**How are you currently feeding your baby?**						
**Breast**	**Mixed**	**Bottle**				
