# Supplementary material for: The ASSIST Study - The BD Odon Device for assisted vaginal birth: a safety and feasibility study
Source: Trials. 2019 Mar 5;20:159. doi: 10.1186/s13063-019-3249-z (PMC6402154; doi:10.1186/s13063-019-3249-z)
Supplement: Supplementary file 1 — Case report forms. Copies of the case report forms for the ASSIST Study. (ZIP 830 kb) [file 13063_2019_3249_MOESM1_ESM.zip › PublicationFiles-supplementary_file_a3R0.pdf]

** PLEASE KEEP THIS FORM IN THE BABY NOTES **

North Bristol NHS
NHS Trust

ASSIST
ASSISTED VAGINAL BIRTH STUDY

Please stick mother's hospital identification label here	Please stick baby's hospital identification label here (if available)

NEONATAL CASE REPORT FORM A

PART 1A: Neonatal short-term outcomes – Obstetrician to complete prior to discharge from CDS			
**Apgar scores**	1 minute	5 minutes	10 minutes
**Umbilical artery pH**		**Umbilical vein pH**	
**Umbilical artery BE**		**Umbilical vein BE**	
**Shoulder dystocia occurred?**	Yes	No	
**Admitted to NICU?**	Yes	No	
**Reason for admission:**	State details here:		

Please affix BD Odon Device ID number label
Ensure additional device ID number label is affixed to the birth notes and maternal pro forma

** PLEASE KEEP THIS FORM IN THE BABY NOTES **

North Bristol NHS NHS Trust

ASSIST ASSISTED VAGINAL BIRTH STUDY

Please stick mother's hospital identification label here	Please stick baby's hospital identification label here (if available)

**PART 1B – to be completed by midwife at 2 and 6 hours of age**			
**NIPS Scoring Tool (please circle relevant score)**		**Score at 2 hours old**	**Score at 6 hours old**
**Facial expression**	Relaxed (restful face, neutral expression)	0	0
	Grimace (tight facial muscles, furrowed brow, chin, jaw)	1	1
**Cry**	No cry (quiet, not crying)	0	0
	Whimper (mild moaning, intermittent)	1	1
	Vigorous crying (loud scream, shrill, continuous). If infant is intubated, score silent cry based on facial movement.	2	2
**Breathing pattern**	Relaxed (usual pattern for this infant)	0	0
	Change in breathing (irregular, faster than usual, gagging, breath holding)	1	1
**Arms**	Relaxed (no muscular rigidity, occasional random movements of arms)	0	0
	Flexed/extended (tense, straight arms, rigid and/or rapid extension, flexion)	1	1
**Legs**	Relaxed (no muscular rigidity, occasional random leg movements)	0	0
	Flexed/Extended (tense, straight legs, rigid and/or rapid extension, flexion)	1	1
**State of Arousal**	Sleeping/Awake (quiet, peaceful, sleeping or alert and settled)	0	0
	Fussy (alert, restless and thrashing)	1	1
**Total score (>4 = severe pain)**			
**Time of score**			
**Name of person who made the NIPS assessment**			

** PLEASE KEEP THIS FORM IN THE BABY NOTES **

North Bristol NHS Trust

ASSIST ASSISTED VAGINAL BIRTH STUDY

Please stick mother's hospital identification label here	Please stick baby's hospital identification label here (if available)

**PART 2: Neonatal medium-term outcomes – to be completed by practitioner performing NIPE check**					
**Date and time NIPE performed**	__ __ / __ __ / __ __ __ __:__ __				
**Admitted to NICU at any point up to the present?**	Yes		No		
**Reason for admission:**	State details:				
**Has the baby had at least one feed up to the present?**	Yes		No		
**Have any neonatal injuries been diagnosed?**	Yes		No		
**If yes, please circle if any of the following have been diagnosed at the time of the NIPE check**					
**Soft tissue trauma**	Bruise	Scalp injury	Facial injury	Pressure necrosis of skin or fat	Other (describe)
**Vascular injury**	Haemorrhage	Cephalohaematoma	Subaponeurotic haemorrhage		
**Skeletal injury**	Bony fracture	Other (describe)			
**Intracranial injury**	Cerebral contusion	Other (describe)			
**Other neonatal injury**	Please describe:				

**Initial Adverse Event Reporting Tool *see appendix X**<br>**Member of research team to complete (photocopy p.1,2,3 to sponsor)**	
**Event Description**	TICK HERE **IF THIS IS A RED EVENT:** ☐
**Situation**	
**Background**	
**Action**	
**Assessment undertaken by:**	**Name:**<br>**Signature:**<br>**Date:**

** PLEASE KEEP THIS FORM IN THE BABY NOTES **

North Bristol NHS
NHS Trust

ASSIST
ASSISTED VAGINAL BIRTH STUDY

Please stick mother's hospital identification label here	Please stick baby's hospital identification label here (if available)

**PART 3: Neonatal long-term outcomes – to be completed by Paediatrician performing outcome screening at 28 days of birth**						
**Date outcome screening performed**	__ __/__ __/__ __					
**Admitted to NICU at any point?**	No	Yes	Date and time admitted: __ __/__ __/__ __ __ __:__ __ Date and time discharged: __ __/__ __/__ __ __ __:__ __			
**Reason for admission:**	State details:					
**Have any neonatal injuries been diagnosed?**	Yes		No			
**If yes, please circle if any of the following have been diagnosed**						
**Soft tissue trauma**	Bruise	Scalp injury	Facial injury	Pressure necrosis of skin or fat	Other (describe):	
**Vascular injury**	Haemorrhage	Cephalohaematoma	Subaponeurotic haemorrhage			
**Skeletal injury**	Bony fracture	Other (describe):				
**Intracranial injury**	Cerebral contusion	Other (describe):				
**Other injury**	Please describe:					

** PLEASE KEEP THIS FORM IN THE BABY NOTES **

North Bristol NHS
NHS Trust

ASSIST
ASSISTED VAGINAL BIRTH STUDY

Please stick mother's hospital identification label here	Please stick baby's hospital identification label here (if available)

**Other neonatal outcomes (please circle)**		
**Was a neurological injury still present at 28 days old?**	Yes	No
**Did the baby have a seizure?**	Yes	No
**Did the baby require phototherapy for jaundice contributed to by bruising?**	Yes	No
**Did the baby have anaemia requiring transfusion?**	Yes	No
**Did the baby require therapeutic cooling for neonatal encephalopathy?**	Yes	No
**Did the baby suffer from organ failure?**	Yes	No
**Did the baby die in the first 28 days after birth?**	Yes	No
**If yes to any of the above please provide details below:**		
