# Supplementary material for: The ASSIST Study - The BD Odon Device for assisted vaginal birth: a safety and feasibility study
Source: Trials. 2019 Mar 5;20:159. doi: 10.1186/s13063-019-3249-z (PMC6402154; doi:10.1186/s13063-019-3249-z)
Supplement: Supplementary file 1 — Case report forms. Copies of the case report forms for the ASSIST Study. (ZIP 830 kb) [file 13063_2019_3249_MOESM1_ESM.zip › PublicationFiles-supplementary_file_a4R0.pdf]

NHS North Bristol NHS Trust

ASSIST ASSISTED VAGINAL BIRTH STUDY

The BD Odon Device for assisted vaginal birth: a safety and feasibility study
The ASSIST Study

Antenatal Health Questionnaire

English version for the UK

**Participant Name:**	
**Study number:**	
**Date of completion:**	
**Patient contact number:**	

UK (English) © 2009 EuroQol Group EQ-5D™ is a trade mark of the EuroQol Group

NHS North Bristol NHS Trust

ASSIST ASSISTED VAGINAL BIRTH STUDY

Under each heading, please tick the ONE box that best describes your health TODAY.

MOBILITY

I have no problems in walking about	❑
I have slight problems in walking about	❑
I have moderate problems in walking about	❑
I have severe problems in walking about	❑
I am unable to walk about	❑

SELF-CARE

I have no problems washing or dressing myself	❑
I have slight problems washing or dressing myself	❑
I have moderate problems washing or dressing myself	❑
I have severe problems washing or dressing myself	❑
I am unable to wash or dress myself	❑

USUAL ACTIVITIES *(e.g. work, study, housework, family or leisure activities)*

I have no problems doing my usual activities	❑
I have slight problems doing my usual activities	❑
I have moderate problems doing my usual activities	❑
I have severe problems doing my usual activities	❑
I am unable to do my usual activities	❑

PAIN / DISCOMFORT

I have no pain or discomfort	❑
I have slight pain or discomfort	❑
I have moderate pain or discomfort	❑
I have severe pain or discomfort	❑
I have extreme pain or discomfort	❑

ANXIETY / DEPRESSION

I am not anxious or depressed	❑
I am slightly anxious or depressed	❑
I am moderately anxious or depressed	❑
I am severely anxious or depressed	❑
I am extremely anxious or depressed	❑

UK (English) © 2009 EuroQol Group EQ-5D™ is a trade mark of the EuroQol Group

NHS North Bristol NHS Trust

ASSIST ASSISTED VAGINAL BIRTH STUDY

The best health you can imagine

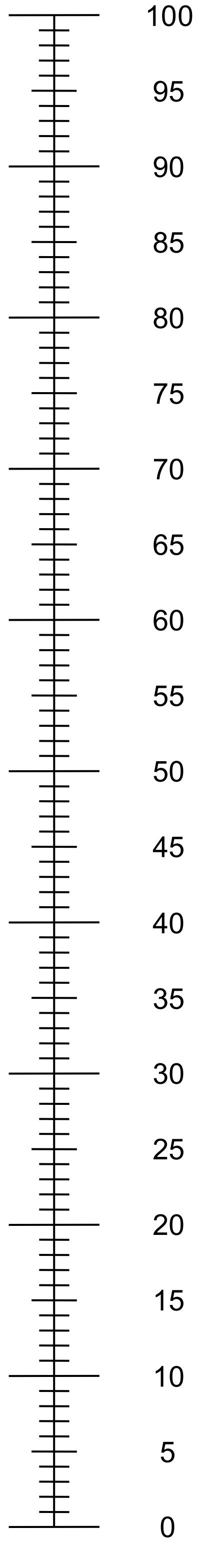

The worst health you can imagine

- We would like to know how good or bad your health is TODAY.
- This scale is numbered from 0 to 100.
- 100 means the best health you can imagine.
  0 means the worst health you can imagine.
- Mark an X on the scale to indicate how your health is TODAY.
- Now, please write the number you marked on the scale in the box below.

YOUR HEALTH TODAY = [ ]

UK (English) © 2009 EuroQol Group EQ-5D™ is a trade mark of the EuroQol Group
