# Supplementary material for: The ASSIST Study - The BD Odon Device for assisted vaginal birth: a safety and feasibility study
Source: Trials. 2019 Mar 5;20:159. doi: 10.1186/s13063-019-3249-z (PMC6402154; doi:10.1186/s13063-019-3249-z)
Supplement: Supplementary file 2 — Operator training for the ASSIST Study. Details of the training programme operators received prior to study start. (DOCX 73 kb) [file 13063_2019_3249_MOESM2_ESM.docx]

**Operator Training for the ASSIST Study**

All staff undertaking assisted vaginal births as part of this study will attend a half-day training session. This will include an instructional video on the use of the BD Odon Device and intensive 1:1 practical teaching of how to use the BD Odon Device on a high-fidelity pelvic simulator. The training is based on the Royal College of Obstetricians and Gynaecologists training course for instrumental birth, ROBuST (17) and the findings of the extensive human factor studies conducted using the BD Odon Device .

These half-day training sessions will be repeated for any new staff and if any applicable amendments are made to the study protocol, to ensure that all staff remain up to date.

Should any practitioner be found during interim analyses to be performing with a success rate deemed to be insufficient they will be re-training in both the BD Odon Device training package and ROBuST.

All members of the research team (i.e. those gaining consent, administering the study and performing AVBs) will hold a valid Good Clinical Practice (GCP) certificate.
