# Supplementary material for: The ASSIST Study - The BD Odon Device for assisted vaginal birth: a safety and feasibility study
Source: Trials. 2019 Mar 5;20:159. doi: 10.1186/s13063-019-3249-z (PMC6402154; doi:10.1186/s13063-019-3249-z)
Supplement: Supplementary file 3 — Schedule of review of data collected in the ASSIST Study. Details of the Sponsor/TSC/IDMC reviews. (DOCX 96 kb) [file 13063_2019_3249_MOESM3_ESM.docx]

**Schedule of review of data collected in ASSIST Study**

**Overview**

Three levels of review will be undertaken during the ASSIST Study; continuous and scheduled reviews and interim analyses. The planned frequency and timing of these is summarised in Table 1, however meeting schedules will be proportionate to the data outcomes and the Trial Steering Committee (TSC) may recommend additional reviews by the Independent Data Monitoring Committee (IDMC) if deemed appropriate.

**Table 1**. Schedule of reviews and analyses within the ASSIST Study

| **Type of review** | **By whom** | **Frequency** |
| --- | --- | --- |
| Continuous review | TMG & Sponsor | Every birth |
| Quinitial review | TMG & Sponsor | Every 5^th^ birth |
| Interim analysis | DMC, TSC & Sponsor | After 20^th^ birth |
| Final analysis | DMC, TSC & Sponsor | After 40^th^ birth |

**Continuous review**

Following every birth, all immediate post-birth outcomes will be reported to the Sponsor with 24 hours of the assisted birth. There are key amber and red outcomes that will be specifically reported on (table 2). The trial will be immediately suspended if one red adverse outcome is reported.

**Table 2.** Log of key outcomes for the ASSIST Study

| **Maternal Outcomes** |
| --- |
| PPH > 3000ml |
| 3^rd^ or 4^th^ degree tear |
| Ischio-rectal fossa defect |
| Cervical tear requiring suturing |
| Maternal death |
| **Neonatal Outcomes** |
| Apgar <7 at 5 minutes |
| Jaundice requiring phototherapy |
| Admission to NICU |
| Pressure necrosis of skin or fat |
| Neonatal death |
| **Device Outcomes** |
| Failure to complete delivery with Odon Device |
| Failure to apply the Odon device |
| Failed inflation of air chamber |
| Failed deflation of air chamber |
| Failure to remove Odon device resulting in a caesarean section |

Amber adverse outcomes are expected to occur during the study period. These are outcomes that are associated with assisted vaginal birth and we are aware that many of these outcomes will occur during the study period. The continuous review process will enable early identification (and potential for rectification) of any adverse outcomes.

**Quinitial review**

Following every fifth birth, a descriptive summary of selected outcomes from both the most recent five births and all births to date will be generated by the Trial Management Group and shared with the Sponsor.

If two (of the same) key adverse (amber) outcomes (shown in table 2) are reported within any sequential five births an Root Cause Analysis will be undertaken and the trial may be paused in discussion with the Sponsor. Following the review, the TSC will make a recommendation to the Sponsor which will then decide whether to continue, revise or stop the trial.

**Interim and final analyses**

All study data will be reviewed after the 20^th^ birth (interim analysis) and 40^th^ birth (final analysis). Analyses will be presented to the IDMC who will provide a report to the TSC and Sponsor.
