# Supplementary material for: The ASSIST Study - The BD Odon Device for assisted vaginal birth: a safety and feasibility study
Source: Trials. 2019 Mar 5;20:159. doi: 10.1186/s13063-019-3249-z (PMC6402154; doi:10.1186/s13063-019-3249-z)
Supplement: Supplementary file 4 — Grant agreement. Summary agreement and signature page from funder. (PDF 147 kb) [file 13063_2019_3249_MOESM4_ESM.pdf]

BILL & MELINDA GATES foundation

**GRANT AGREEMENT**
Investment ID OPP1184825

**AGREEMENT SUMMARY & SIGNATURE PAGE**

| **GRANTEE INFORMATION** | |
|---|---|
| Name: | PROMPT Maternity Foundation |
| Tax Status: | Public Charity equivalent pursuant to U.S. IRC § 509(a)(1) or 509(a)(2)<br>You confirm that the above information is correct and agree to notify the Foundation immediately of any change. |
| Mailing Address: | Department of Women's Health The Chilterns<br>Southmead Hospital<br>Bristol  BS10 5NB<br>United Kingdom |
| Primary Contact: | Joanna Crofts, Consultant, jocrofts@me.com |

| **FOUNDATION INFORMATION** | |
|---|---|
| Mailing Address: | P. O. Box 23350, Seattle, WA  98102, U.S.A. |
| Primary Contact: | Steven Kern, Deputy Director, Quantitative Sciences,<br>Steven.Kern@gatesfoundation.org |

| **AGREEMENT INFORMATION** | |
|---|---|
| Title: | Comparing the BD Odon Device with Kiwi Vacuum - a multi-country RCT |
| "Charitable Purpose": | To create new processes and technologies proven and available to decrease maternal mortality and morbidity |
| "Start Date": | Date of last signature |
| "End Date": | March 31, 2020 |
| This Agreement includes and incorporates by this reference: | This Agreement Summary & Signature Page and:<br>● Grant Amount and Reporting & Payment Schedule (Attachment A)<br>● Terms and Conditions (Attachment B)<br>● Proposal Narrative (date submitted February 8, 2018)<br>● Results Framework and Tracker (date submitted October 11, 2017)<br>● Budget (date submitted February 8, 2018) |

**THIS AGREEMENT** is between PROMPT Maternity Foundation ("*You*" or "*Grantee*") and the Bill & Melinda Gates Foundation ("*Foundation*"), and is effective as of the date of last signature. Each party to this Agreement may be referred to individually as a "*Party*" and together as the "*Parties.*" As a condition of this grant, the Parties enter into this Agreement by having their authorized representatives sign below.

**BILL & MELINDA GATES FOUNDATION**

DocuSigned by:
Hugh Chang
67CD861E697A44A...

By: Hugh Chang
Title: Interim Director, MNCH

February 22, 2018

Date

**PROMPT MATERNITY FOUNDATION**

By:
Title:

Date

**GRANT AGREEMENT**
Investment ID OPP1184825

**ATTACHMENT A**
GRANT AMOUNT AND REPORTING & PAYMENT SCHEDULE

**GRANT AMOUNT**
The Foundation will pay You the total grant amount specified in the Reporting & Payment Schedule below. The Foundation's Primary Contact must approve in writing any Budget cost category change of more than 10%.

**REPORTING & PAYMENT SCHEDULE**
Payments are subject to Your compliance with this Agreement, including Your achievement, and the Foundation's approval, of any applicable targets, milestones, and reporting deliverables required under this Agreement. The Foundation may, in its reasonable discretion, modify payment dates or amounts and will notify You of any such changes in writing.

**REPORTING**
You will submit reports according to the Reporting & Payment Schedule using the Foundation's templates or forms, which the Foundation will make available to You and which may be modified from time to time. For a progress or final report to be considered satisfactory, it must demonstrate meaningful progress against the targets or milestones for that investment period. If meaningful progress has not been made, the report should explain why not and what adjustments You are making to get back on track. Please notify the Foundation's Primary Contact if You need to add or modify any targets or milestones. The Foundation must approve any such changes in writing. You agree to submit other reports the Foundation may reasonably request.

| **REPORTING & PAYMENT SCHEDULE** | | | | |
|---|---|---|---|---|
| *Investment Period* | *Target, Milestone, or Reporting Deliverable* | *Due By* | *Payment Date* | *Payment Amount (U.S.$)* |
| | Countersigned Agreement | | Within 15 days after receipt of countersigned Agreement | $1,210,694 |
| Start Date through March 31, 2019 | Progress Report | April 2019 | May 2019 | $826,475 |
| Start Date through End Date | Final Report | Within 90 days of End Date | | |
| | | | **Total Grant Amount** | **$2,037,169** |

**GRANT AGREEMENT**
Investment ID OPP1184825

**ATTACHMENT B**
TERMS & CONDITIONS

This Agreement is subject to the following terms and conditions.

## PROJECT SUPPORT

### PROJECT DESCRIPTION AND CHARITABLE PURPOSE

The Foundation is awarding You this grant to carry out the project described in the Proposal Narrative and Results Framework and Tracker (collectively, "*Project*") in order to further the Charitable Purpose. The Foundation, in its discretion, may approve in writing any request by You to make non-material changes to the Proposal Narrative and/or Results Framework and Tracker.

## MANAGEMENT OF FUNDS

### USE OF FUNDS

You may not use funds provided under this Agreement ("*Grant Funds*") for any purpose other than the Project. You may not use Grant Funds to reimburse any expenses You incurred prior to the Start Date. At the Foundation's request, You will repay any portion of Grant Funds and/or Income used or committed in material breach of this Agreement, as determined by the Foundation in its discretion.

### INVESTMENT OF FUNDS

You must invest Grant Funds in highly liquid investments with the primary objective of preservation of principal (e.g., interest-bearing bank accounts or a registered money market mutual fund) so that the Grant Funds are available for the Project. Together with any progress or final reports required under this Agreement, You must report the amount of any currency conversion gains (or losses) and the amount of any interest, or other income generated by the Grant Funds (collectively, *"Income"*). Any Income must be used for the Project.

## GLOBAL ACCESS

### GLOBAL ACCESS COMMITMENT

You will conduct and manage the Project and the Funded Developments in a manner that ensures Global Access. Your Global Access commitments will survive the term of this Agreement. "*Funded Developments*" means the products, services, processes, technologies, materials, software, data, other innovations, and intellectual property resulting from the Project (including modifications, improvements, and further developments to Background Technology). "*Background Technology*" means any and all products, services, processes, technologies, materials, software, data, or other innovations, and intellectual property created by You or a third party prior to or outside of the Project used as part of the Project. "*Global Access*" means: (a) the knowledge and information gained from the Project will be promptly and broadly disseminated; and (b) the Funded Developments will be made available and accessible at an affordable price (i) to people most in need within developing countries, or (ii) in support of the U.S. educational system and public libraries, as applicable to the Project.

### LICENSE TO THE FOUNDATION

For the purpose of achieving Global Access, You grant the Foundation a nonexclusive, perpetual, irrevocable, worldwide, royalty-free, fully paid up, sublicensable license to: make, use, sell, offer to sell, import, distribute, copy, modify, create derivative works, publicly perform and display the Funded Developments and any Background Technology incorporated into a Funded Development or required to use a Funded Development. In the event You demonstrate to the satisfaction of the Foundation that Global Access can best be achieved without such a license (or a license of different scope) the Foundation and You will make good faith efforts to modify or terminate this license, as appropriate.

### PUBLICATION

Consistent with Your Global Access commitments, if the Project description specifies Publication or Publication is otherwise requested by the Foundation, You will seek prompt Publication of any Funded

Developments consisting of data and results. "*Publication*" means publication in a peer-reviewed journal or other method of public dissemination specified in the Project description or otherwise approved by the Foundation in writing. Publication may be delayed for a reasonable period for the sole purpose of seeking patent protection, provided the patent application is drafted, filed, and managed in a manner that best furthers Global Access. If You seek Publication in a peer-reviewed journal, such Publication shall be under "open access" terms and conditions consistent with the Foundation's Open Access Policy available at: www.gatesfoundation.org/How-We-Work/General-Information/Open-Access-Policy, which may be modified from time to time. Nothing in this section shall be construed as requiring Publication in contravention of any applicable ethical, legal, or regulatory requirements. You will mark any Funded Development subject to this clause with the appropriate notice or attribution, including author, date and copyright (*e.g.*, © 20<> <Name>).

### INTELLECTUAL PROPERTY REPORTING

During the term of this Agreement and for 5 years after, You will submit upon request annual intellectual property reports related to the Funded Developments, Background Technology, and any related agreements using the Foundation's templates or forms, which the Foundation may modify from time to time.

## SUBGRANTS AND SUBCONTRACTS

### SUBGRANTS AND SUBCONTRACTS

You have the exclusive right to select subgrantees and subcontractors to assist with the Project.

### SCHOLARSHIPS AND FELLOWSHIPS

You will have sole discretion over Your selection of any scholarship and fellowship recipients under this Agreement and must conduct the selection process independently of the Foundation.

### TRAVEL STIPENDS AND CONFERENCE FEES

You will have sole discretion over Your selection of any recipients of travel stipends or conference expense reimbursements under this Agreement and must conduct the selection process independently of the Foundation. Foundation trustees and employees are not eligible to receive travel stipends or conference expense reimbursements.

### RESPONSIBILITY FOR OTHERS

You are responsible for (a) all acts and omissions of any of Your trustees, directors, officers, employees, subgrantees, subcontractors, contingent workers, agents, and affiliates assisting with the Project, and (b) ensuring their compliance with the terms of this Agreement.

## PROHIBITED ACTIVITIES

### ANTI-TERRORISM

You will not use funds provided under this Agreement, directly or indirectly, in support of activities (a) prohibited by U.S. laws relating to combating terrorism; (b) with persons on the List of Specially Designated Nationals (www.treasury.gov/sdn) or entities owned or controlled by such persons; or (c) in or with countries or territories against which the U.S. maintains comprehensive sanctions (currently, Cuba, Iran, Syria, North Korea, and the Crimea Region of Ukraine), including paying or reimbursing the expenses of persons from such countries or territories, unless such activities are fully authorized by the U.S. government under applicable law and specifically approved by the Foundation in its sole discretion.

### ANTI-CORRUPTION; ANTI-BRIBERY

You will not offer or provide money, gifts, or any other things of value directly or indirectly to anyone in order to improperly influence any act or decision relating to the Foundation or the Project, including by assisting any party to secure an improper advantage. Training and information on compliance with these requirements are available at www.learnfoundationlaw.org.

### LOBBYING AND ELECTIONEERING PROHIBITION

You may not use Grant Funds to influence the outcome of any election for public office or to carry on any voter registration drive. You acknowledge that the Foundation has not earmarked Grant Funds to support lobbying activities or to otherwise support attempts to influence legislation. Activities will be conducted

consistent with the private foundation lobbying rules and exceptions under Internal Revenue Code Section 4945 and related regulations. You confirm that the Budget (or the combined project budget if there are multiple funders) accurately reflects that You will expend at least the amount of the Grant Funds on (a) non-lobbying activities in the project year, or (b) for multiple year projects, the total non-lobbying portion of the project.

### OTHER LOBBYING, GIFT, AND ETHICS RULES
You agree to comply with any national, state, local, or other lobbying, gift, and ethics rules applicable to the Project. The Foundation is not retaining or employing You to engage in lobbying activities.

## PUBLICITY

### PUBLICITY BY THE FOUNDATION
The Foundation may include information about the award of this grant, including Your name, in its periodic public reports and may make such information available on its website and as part of press releases, public reports, speeches, newsletters, tax returns, and other public disclosures.

### PUBLICITY BY YOU
You must obtain the Foundation's prior written approval before: (a) issuing a press release or other public announcement regarding this grant; and (b) any other public use of the Foundation's name or logo. Please email Your request to: grantee.comms@gatesfoundation.org two weeks in advance to provide the Foundation an opportunity to review and comment. Detailed guidelines are available at:
www.gatesfoundation.org/grantseeker/documents/guidelines_communications_for_grantees.doc.

### PUBLICITY BY OTHERS
You and Your subgrantees, subcontractors, contingent workers, agents, or affiliates may not state or otherwise imply to third parties that the Foundation directly funds or otherwise endorses their activities.

## OTHER

### COMPLIANCE WITH LAWS
In carrying out the Project, You will comply with all applicable laws, regulations, and rules and will not infringe, misappropriate, or violate the intellectual property, privacy, or publicity rights of any third party.

### COMPLIANCE WITH REQUIREMENTS
You will conduct, control, manage, and monitor the Project in compliance with all applicable ethical, legal, regulatory, and safety requirements, including applicable international, national, local, and institutional standards ("*Requirements*"). You will obtain and maintain all necessary approvals, consents, and reviews before conducting the applicable activity. As a part of Your annual progress report to the Foundation, You must report whether the Project activities were conducted in compliance with all Requirements.

If the Project involves:
a. any protected information (including personally identifiable, protected health, or third-party confidential), You will not disclose this information to the Foundation without obtaining the Foundation's prior written approval and all necessary consents to disclose such information;
b. children or vulnerable subjects, You will obtain any necessary consents and approvals unique to these subjects; and/or
c. any trial involving human subjects, You will adhere to current Good Clinical Practice as defined by the International Council on Harmonisation (ICH) E-6 Standards (or local regulations if more stringent) and will obtain applicable trial insurance.

Any activities by the Foundation in reviewing documents and providing input or funding does not modify Your responsibility for determining and complying with all Requirements for the Project.

### RELIANCE
You acknowledge that the Foundation is relying on the information You provide in reports and during the course of any due diligence conducted prior to the Start Date and during the term of this Agreement. You represent that the Foundation may continue to rely on this information and on any additional information You provide regarding activities, progress, and Funded Developments.

**INDEMNIFICATION**

If the Project involves clinical trials, trials involving human subjects, post-approval studies, field trials involving genetically modified organisms, experimental medicine, or the provision of medical/health services ("*Indemnified Activities*"), You will indemnify, defend, and hold harmless the Foundation and its trustees, employees, and agents ("*Indemnified Parties*") from and against any and all demands, claims, actions, suits, losses, damages (including property damage, bodily injury, and wrongful death), arbitration and legal proceedings, judgments, settlements, or costs or expenses (including reasonable attorneys' fees and expenses) (collectively, "*Claims*") arising out of or relating to the acts or omissions, actual or alleged, of You or Your employees, subgrantees, subcontractors, contingent workers, agents, and affiliates with respect to the Indemnified Activities. You agree that any activities by the Foundation in connection with the Project, such as its review or proposal of suggested modifications to the Project, will not modify or waive the Foundation's rights under this paragraph. An Indemnified Party may, at its own expense, employ separate counsel to monitor and participate in the defense of any Claim. Your indemnification obligations are limited to the extent permitted or precluded under applicable federal, state or local laws, including federal or state tort claims acts, the Federal Anti-Deficiency Act, state governmental immunity acts, or state constitutions. Nothing in this Agreement will constitute an express or implied waiver of Your governmental and sovereign immunities, if any.

**INSURANCE**

You will maintain insurance coverage sufficient to cover the activities, risks, and potential omissions of the Project in accordance with generally-accepted industry standards and as required by law. You will ensure Your subgrantees and subcontractors maintain insurance coverage consistent with this section.

## TERM AND TERMINATION

**TERM**

This Agreement commences on the Start Date and continues until the End Date, unless terminated earlier as provided in this Agreement. The Foundation, in its discretion, may approve in writing any request by You for a no-cost extension, amend the End, and adjust any affected reporting requirements.

**TERMINATION**

The Foundation may modify, suspend, or discontinue any payment of Grant Funds or terminate this Agreement if: (a) the Foundation is not reasonably satisfied with Your progress on the Project; (b) there are significant changes to Your leadership or other factors that the Foundation reasonably believes may threaten the Project's success; (c) there is a change in Your control; (d) there is a change in Your tax status; or (e) You fail to comply with this Agreement.

**RETURN OF FUNDS**

Any Grant Funds that have not been used for, or committed to, the Project upon expiration or termination of this Agreement must be (a) returned promptly to the Foundation, (b) applied to another Foundation-funded project (current or under consideration), or (c) applied to another mutually-agreed upon charitable purpose, as directed in writing by the Foundation. Any Income that has not been used for, or committed to, the Project must be either applied to another Foundation-funded project (current or under consideration) or applied to another mutually-agreed upon charitable purpose, as directed in writing by the Foundation.

**MONITORING, REVIEW, AND AUDIT**

The Foundation may monitor and review Your use of the Grant Funds, performance of the Project, and compliance with this Agreement, which may include onsite visits to assess Your organization's governance, management and operations, discuss Your program and finances, and review relevant financial and other records and materials. In addition, the Foundation may conduct audits, including onsite audits, at any time during the term of this Agreement, and within four years after Grant Funds have been fully spent. Any onsite visit or audit shall be conducted at the Foundation's expense, following prior written notice, during normal business hours, and no more than once during any 12-month period.

**INTERNAL OR THIRD PARTY AUDIT**

If during the term of this Agreement You are audited by your internal audit department or by a third party, You will provide the audit report to the Foundation upon request, including the management letter and a detailed plan for remedying any deficiencies observed ("*Remediation Plan*"). The Remediation Plan must

include (a) details of actions You will take to correct any deficiencies observed, and (b) target dates for successful completion of the actions to correct the deficiencies.

**RECORD KEEPING**
You will maintain complete and accurate accounting records and copies of any reports submitted to the Foundation relating to the Project. You will retain such records and reports for 4 years after Grant Funds have been fully spent. At the Foundation's request, You will make such records and reports available to enable the Foundation to monitor and evaluate how Grant Funds have been used or committed.

**SURVIVAL**
A Party's obligations under this Agreement will be continuous and survive expiration or termination of this Agreement as expressly provided in this Agreement or otherwise required by law or intended by their nature.

## GENERAL

**ENTIRE AGREEMENT AND AMENDMENTS**
This Agreement contains the entire agreement of the Parties and supersedes all prior and contemporaneous agreements concerning its subject matter. Except as specifically permitted in this Agreement, no modification, amendment, or waiver of any provision of this Agreement will be effective unless in writing and signed by authorized representatives of both Parties.

**NOTICES AND APPROVALS**
Written notices, requests, and approvals under this Agreement must be delivered by mail or email to the other Party's primary contact specified on the Agreement Summary & Signature Page, or as otherwise directed by the other Party.

**SEVERABILITY**
Each provision of this Agreement must be interpreted in a way that is enforceable under applicable law. If any provision is held unenforceable, the rest of the Agreement will remain in effect.

**ASSIGNMENT**
You may not assign, or transfer by operation of law or court order, any of Your rights or obligations under this Agreement without the Foundation's prior written approval. This Agreement will bind and benefit any permitted successors and assigns.

**COUNTERPARTS AND ELECTRONIC SIGNATURES**
Except as may be prohibited by applicable law or regulation, this Agreement and any amendment may be signed in counterparts, by facsimile, PDF, or other electronic means, each of which will be deemed an original and all of which when taken together will constitute one agreement. Facsimile and electronic signatures will be binding for all purposes.
