# Supplementary material for: The ASSIST Study - The BD Odon Device for assisted vaginal birth: a safety and feasibility study
Source: Trials. 2019 Mar 5;20:159. doi: 10.1186/s13063-019-3249-z (PMC6402154; doi:10.1186/s13063-019-3249-z)
Supplement: Supplementary file 6 — Ethics Committee Approval for the ASSIST Study format. Letter from the South Central Berkshire Ethics Committee. (PDF 372 kb) [file 13063_2019_3249_MOESM6_ESM.pdf]

South Central - Berkshire Research Ethics Committee
Bristol REC Centre
Whitefriars
Level 3, Block B
Lewins Mead
Bristol
BS1 2NT

Telephone: 020 7104 8057

Please note: This is the favourable opinion of the REC only and does not allow you to start your study at NHS sites in England until you receive HRA Approval

28 June 2018

Professor Timothy Draycott
Professor of Obstetrics
North Bristol NHS Trust
The Chilterns
Southmead Hospital
Bristol
BS10 5NB

Dear Professor Draycott

**Study title:**	**The BD Odon Device for assisted vaginal birth: a safety and feasibility study**
**REC reference:**	**18/SC/0344**
**Protocol number:**	**16.7**
**IRAS project ID:**	**213604**

The Research Ethics Committee reviewed the above application at the meeting held on 19 June 2018.

Thank you for involving me in the early planning stages of this study; it was an interesting experience for me but, more importantly it contributed to ensuring that ethics was at the heart of the study and that the women involved would be given meaningful roles in the research rather than being passive participants.

We plan to publish your research summary wording for the above study on the HRA website, together with your contact details. Publication will be no earlier than three months from the date of this favourable opinion letter. The expectation is that this information will be published for all studies that receive an ethical opinion but should you wish to provide a substitute contact point, wish to make a request to defer, or require further information, please contact hra.studyregistration@nhs.net outlining the reasons for your request.

Under very limited circumstances (e.g. for student research which has received an unfavourable opinion), it may be possible to grant an exemption to the publication of the study.

Ethical opinion

The members of the Committee present gave a favourable ethical opinion of the above research on the basis described in the application form, protocol and supporting documentation, subject to the conditions specified below. .

Conditions of the favourable opinion

The REC favourable opinion is subject to the following conditions being met prior to the start of the study.

1. Please add the following to the PIS:

   a) Please add to the summary PIS that further information would be available at or after 28 weeks. The IRAS form mentioned a maternity app but this was not referenced in this summary PIS, please add this.

   b) Please add that in a situation of foetal compromise the study device would not be used as this situation was urgent.

   c) Please add, under the skin to skin contact and delayed cord clamping section, a proviso covering emergency situations where this might not be possible.

   d) Under 'Will participating take up lots of time' the PIS says 'No'. However, there will be additional time required and contact with research midwives both on the ward and telephone calls at home, plus questionnaires. Please remove 'No' and rephrase this.

   e) Add latex sensitivity as an exclusion criterion to both the PIS and protocol.

   f) The Committee asked for further information to be added to the PIS to state the role of the midwife in obtaining consent; they would have the opportunity to give informal feedback on the process of consent.

You should notify the REC once all conditions have been met (except for site approvals from host organisations) and provide copies of any revised documentation with updated version numbers. Revised documents should be submitted to the REC electronically from IRAS. The REC will acknowledge receipt and provide a final list of the approved documentation for the study, which you can make available to host organisations to facilitate their permission for the study. Failure to provide the final versions to the REC may cause delay in obtaining permissions.

<u>Management permission must be obtained from each host organisation prior to the start of the study at the site concerned.</u>

Management permission should be sought from all NHS organisations involved in the study in accordance with NHS research governance arrangements. Each NHS organisation must

confirm through the signing of agreements and/or other documents that it has given permission for the research to proceed (except where explicitly specified otherwise).

Guidance on applying for HRA and HCRW Approval (England and Wales)/ NHS permission for research is available in the Integrated Research Application System, at www.hra.nhs.uk or at http://www.rdforum.nhs.uk.

Where a NHS organisation's role in the study is limited to identifying and referring potential participants to research sites ("participant identification centre"), guidance should be sought from the R&D office on the information it requires to give permission for this activity.

For non-NHS sites, site management permission should be obtained in accordance with the procedures of the relevant host organisation.

Sponsors are not required to notify the Committee of management permissions from host organisations.

Registration of Clinical Trials

All clinical trials (defined as the first four categories on the IRAS filter page) must be registered on a publically accessible database. This should be before the first participant is recruited but no later than 6 weeks after recruitment of the first participant.

There is no requirement to separately notify the REC but you should do so at the earliest opportunity e.g. when submitting an amendment. We will audit the registration details as part of the annual progress reporting process.

To ensure transparency in research, we strongly recommend that all research is registered but for non-clinical trials this is not currently mandatory.

If a sponsor wishes to request a deferral for study registration within the required timeframe, they should contact hra.studyregistration@nhs.net. The expectation is that all clinical trials will be registered, however, in exceptional circumstances non registration may be permissible with prior agreement from the HRA. Guidance on where to register is provided on the HRA website.

Notice of no objection must be obtained from the Medicines and Healthcare products Regulatory Agency (MHRA).

The sponsor is asked to provide the Committee with a copy of the notice from the MHRA, either confirming no objection or giving grounds for objection, as soon as this is available.

It is the responsibility of the sponsor to ensure that all the conditions are complied with before the start of the study or its initiation at a particular site (as applicable).

Ethical review of research sites

NHS Sites

The favourable opinion applies to all NHS sites taking part in the study taking part in the study, subject to management permission being obtained from the NHS/HSC R&D office prior to the start of the study (see "Conditions of the favourable opinion" below).

Non NHS sites

The Committee has not yet completed any site-specific assessment(s) (SSA) for the non-NHS research site(s) taking part in this study. The favourable opinion does not therefore apply to any non-NHS site at present. I will write to you again as soon as an SSA application(s) has been reviewed. In the meantime no study procedures should be initiated at non-NHS sites.

Summary of discussion at the meeting
Ethical issues raised by the Committee in private discussion, together with responses given by the researcher when invited into the meeting

The Chair welcomed the researchers to the meeting and said that there were observers present, who could be removed if necessary. The researchers were happy for the observers to stay.

- **Social or scientific value; scientific design and conduct of the study**

The Committee noted the obvious value of this trial. The device could prove to be better than available alternatives and it could be particularly useful in developing countries.

The Committee was impressed by the use of PPI. Feedback from participating women would continue to influence the trial and some women would be invited to join the advisory group.

The Committee asked whether, in cases of extreme urgency, the trial device would be used or would a standard instrument be used instead.

Dr Croft explained that it was difficult to record this in the PIS but she clarified that it would not be appropriate to use the trial device in an urgent situation.

The Committee asked for this to be added to the PIS as it was clear in the protocol.

The researchers agreed to add this.

The Committee observed that there was confusion around the use of forceps in the study. There was reference to forceps but the Committee asked if they would ever be used in practice. It asked the researchers to clarify this.

With reference to the prospective RCT, Dr Croft explained that the team had considered the alternative devices to which participants could be randomised. The Kiwi ventouse was the most comparable as it was a single use device and had more in common with the trial device than alternatives such as forceps. Dr Croft said that the BD Odon Device vs the forceps was more difficult to do because most women did not want forceps. The use of forceps could remain as a 'backup' (which is effectively normal practice) if either of the trial devices prove unsuccessful.

- **Recruitment arrangements and access to health information, and fair participant selection**

The Committee noted that there was some information regarding the recruitment of doctors, which included a PIS and consent form but apart from a brief mention of recruitment of a subset of women and doctors for interview there was not any further information on how this would be done. There was also mention that the views of midwives would be sought but there was no indication of their inclusion.

Ms Winter stated that the doctor would observe the birth for the qualitative part of the research, i.e. the room experience, the lighting, etc. The midwife interviews would largely focus on feedback surrounding the recruitment process; they would not be contributing to research data.

The Committee asked for further information to be added to the PIS in the form of a simple statement that the midwife seeking consent would be giving general (but not in relation to any individual participant) feedback on the recruitment process.

- **Favourable risk benefit ratio; anticipated benefit/risks for research participants (present and future)**

The Committee asked what would happen if the new device fails.

Dr Croft said that the advice was to use the study device as the first instrument for women consented to the trial, then if this fails the Kiwi ventouse would be used, then finally the forceps. Dr Croft clarified that they would not use forceps in any arm of the prospective RCT.

The Committee questioned whether the researchers needed to make the comparative risks more explicit in the PIS. There was a table in the protocol on page 18 that could be useful to include or adapt. The Committee discussed this and it was agreed that women would know the risks of the standard interventions as they would be addressed in antenatal classes. The ethical challenge was that the participants were being recruited in this study at a time where they did not want to think about risks of birth. The Committee noted that the researchers stated that the device had not been used before and that it was not without risks, however they believed that it was no more risky than the other available options.

- **Care and protection of research participants; respect for potential and enrolled participants' welfare and dignity**

The Committee asked if there would be a follow up of parents after 6 weeks if the baby had died.

Dr Croft confirmed that they would not follow up any parents if the baby had died.

The Committee noted that the brochure instructions included a list of symbols, one of which said 'contains latex'. It asked if the Odon device contained latex, if so then it was suggested that latex sensitivity should be added as an exclusion criteria.

Dr Croft explained that it did contain latex but only on the bulb which was used to inflate the cuff. Researchers were currently having discussions with the manufacturer to change this in the future but for now it would stay the same. Dr Croft agreed to add this as an exclusion criterion for the study.

The Committee noted that the researchers' answer to IRAS A23 was 'No' to topics that could be sensitive. It suggested that this should be 'yes' as there was potential for discussions at the 90 day point to include urinary incontinence.

The researchers agreed with the Committee.

- **Informed consent process and the adequacy and completeness of participant information**

The Committee questioned the consent process as it was mentioned in the application that the research team could approach participants for consent for the first time in the labour

ward. The Committee needed this to be clarified as it was concerned that these participants would be consenting whilst in a vulnerable and emotional state. Could people really consent to anything during labour?

Dr Crofts told the Committee that the usual consent process was that participants would be given all information during antenatal care or if the participant was in early labour and they could agree at this point. This was the same consent process as separate studies that they had undertaken and the decision was always made in early labour or beforehand. Dr Croft said that consenting would take place at 28 weeks so that for most participants they would just be reconfirming consent at the early labour point. The target recruitment was women having their first ever labour and, for the vast majority, consent would have been taken before labour. She assured the Committee that there would be no attempts to recruit any participant in the more advanced stages of labour when they might have had opiates.

The Committee asked for further changes or clarifications to be added to the participant facing information, detailed in the Committee's decision letter.

- **Suitability of the applicant and supporting staff**

The Committee asked about the senior doctors involved in the study and what level they would be. Was there cover when these senior people were not in the hospital and were members of the team trained?

Dr Croft said that the team had not finished training yet as she wanted to do this close to the time of the study start. The senior team were consultant obstetricians or senior level lecturers, so the 'third tier'. The team in this project would always be at levels 5, 6 or 7 and would be the most senior people. Patient safety was at the heart of everything that they were doing. Dr Croft said that there was not 24hr consultant cover, it would usually run from 8am – 8.30pm but there was 24hr tier 5 and above cover, and as a matter of fact, Dr Croft and other consultants were in the hospital a lot.

Other ethical issues were raised and resolved in preliminary discussion before your attendance at the meeting.

Please contact the REC Manager if you feel that the above summary is not an accurate reflection of the discussion at the meeting.

Approved documents

The documents reviewed and approved at the meeting were:

Document	*Version*	*Date*
Copies of advertisement materials for research participants [Transcript of ASSIST Information Video]	1	01 June 2018
Covering letter on headed paper [Covering Letter]	2	04 June 2018
GP/consultant information sheets or letters [ASSIST GP Letter]	1	22 May 2018
Instructions for use of medical device [BD Odon Device IFU]	1	24 November 2017
Interview schedules or topic guides for participants [ASSIST Observational Data Scheme]	v4	03 June 2018
Interview schedules or topic guides for participants [ASSIST Topic Guide (Doctors)]	v2	03 June 2018
Interview schedules or topic guides for participants [Assist Topic Guide (Patients)]	v1	03 June 2018

IRAS Application Form [IRAS_Form_05062018]		05 June 2018
IRAS Application Form XML file [IRAS_Form_05062018]		05 June 2018
IRAS Checklist XML [Checklist_05062018]		05 June 2018
IRAS Checklist XML [Checklist_07062018]		07 June 2018
Letter from funder [Letter from Funder]	1	06 March 2018
Non-validated questionnaire [ASSIST Maternal CRF A]	v5	03 June 2018
Non-validated questionnaire [ASSIST Maternal CRF B]	v2	03 June 2018
Non-validated questionnaire [ASSIST Neonatal CRF]	v3	03 June 2018
Non-validated questionnaire [ASSIST Healthcare Utilisation Form]	v1	03 June 2018
Other [PI CV]	1	03 June 2018
Participant consent form [ASSIST Consent Form]	8	04 June 2018
Participant consent form [ASSIST Consent to audio record]	1	03 June 2018
Participant consent form [ASSIST Eligibility to consent form]	v1	03 June 2018
Participant consent form [ASSIST Doctor consent form]	v2	03 June 2018
Participant information sheet (PIS) [ASSIST PIL]	8	03 June 2018
Participant information sheet (PIS) [ASSIST PIS]	2	03 June 2018
Participant information sheet (PIS) [ASISST Doctor information leaflet]	1	03 June 2018
Research protocol or project proposal [ASSIST Protocol]	16.7	03 June 2018
Summary CV for Chief Investigator (CI) [CI CV]	1	03 June 2018
Validated questionnaire [ASSIST EQ5D5L]	v1	03 June 2018

Membership of the Committee

The members of the Ethics Committee who were present at the meeting are listed on the attached sheet.

The Committee is constituted in accordance with the Governance Arrangements for Research Ethics Committees and complies fully with the Standard Operating Procedures for Research Ethics Committees in the UK.

After ethical review

<u>Reporting requirements</u>

The attached document “After ethical review – guidance for researchers” gives detailed guidance on reporting requirements for studies with a favourable opinion, including:

- Notifying substantial amendments
- Adding new sites and investigators
- Notification of serious breaches of the protocol
- Progress and safety reports
- Notifying the end of the study

The HRA website also provides guidance on these topics, which is updated in the light of changes in reporting requirements or procedures.

User Feedback

The Health Research Authority is continually striving to provide a high quality service to all applicants and sponsors. You are invited to give your view of the service you have received and the application procedure. If you wish to make your views known please use the feedback form available on the HRA website: http://www.hra.nhs.uk/about-the-hra/governance/quality-assurance/

HRA Training

We are pleased to welcome researchers and R&D staff at our training days – see details at http://www.hra.nhs.uk/hra-training/

**18/SC/0344**	**Please quote this number on all correspondence**

With the Committee's best wishes for the success of this project.

Yours sincerely

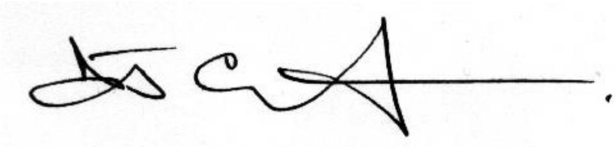

Mr David Carpenter
Chair

E-mail: nrescommittee.southcentral-berkshire@nhs.net

Enclosures:	*List of names and professions of members who were present at the meeting and those who submitted written comments*<br><br>*"After ethical review – guidance for researchers"* [*SL-AR2 for other studies]*
Copy to:	*Ms Helen Lewis-White, North Bristol NHS Trust*<br>*Ms Helen Lewis-White, North Bristol NHS Trust*

South Central - Berkshire Research Ethics Committee

Attendance at Committee meeting on 19 June 2018

Committee Members:

Name	*Profession*	*Present*	*Notes*
Mr David Carpenter	Social Scientist	Yes	
Dr Mike Emanuel	Pharmaceutical Consultant	Yes	
Mr Martin Hopkinson	Director of risk management services	Yes	
Mrs Liz Hunter	Retired Midwife and Clinical Governance Manager	Yes	
Dr Vandana Luthra	R&D Research Co-ordinator	Yes	
Mr Daniel Charles Mace	Retired Corporate Lawyer	Yes	
Mr Richard Merewood	Director	Yes	
Mr Neil Thomas O'Kane	Aviation Safety Consultant	Yes	
Dr Joanne Philpot	Consultant Paediatrician	Yes	
Dr Mike Proven	Coordinator for QA in Research	Yes	
Ms Ann Quinn	Social Worker	Yes	
Dr Deborah Scholey	Regulatory Affairs Consultant	Yes	
Mr Donald Scott-Collett	Lead Pharmacist for Elderly Care, Neuro-rehabilitation, Dermatology and Clinical Governance	Yes	
Dr John Andrew Sutton	Medical Director	No	
Ms Susan Tonks	Senior Research Support Associate	Yes	
Mrs Helen Turner	Clinical Study Manager	No	

Also in attendance:

Name	*Position (or reason for attending)*
Mr Alex Martin	REC Manager
